# Physical health monitoring before commencing regular antipsychotics in a Psychiatric Intensive Care Unit (PICU) - A Quality Improvement project

**DOI:** 10.1192/bjo.2021.501

**Published:** 2021-06-18

**Authors:** Divyanish Divyanish, Afshan Channa

**Affiliations:** Black Country Partnership NHS Foundation Trust

## Abstract

**Aims:**

To compare the practice in a PICU setting against the standard practicing guidelines before commencing antipsychotics with regards to:

1. Physical examination
2. ECG
3. Baseline blood investigations
4. Physical health conditions
5. Family history of medical conditions.

**Method:**

Data were collected from the PICU, Black Country Healthcare NHS Foundation Trust which covers four different hospital sites. 37 patients were admitted in PICU from 1st March 2020 to 30th September 2020, out of which 30 were included. 6 case notes were not available and one patient was admitted twice, thus case notes for only one admission was included in data collection.

The standard guidelines for PICU outline that each admitted patient should have physical examination, vitals monitoring and baseline investigations including routine blood tests and ECG within first 24 hours. The data were collected as per standards retrospectively within two weeks from case notes in health records. Investigations were accessed through electronic information system for current inpatient admission and 12 months prior to the admission to the PICU.

**Result:**

Mean age of the sample (n = 30) was 34.26 years. 37% of patients had physical comorbidities and a family history of medical conditions was documented for only 3% of cases. A large proportion of inpatients (53%) refused to have blood investigations before treatment and only 13% of blood investigations were completed before commencing treatment. Only 7% of patients consented to an ECG prior to commencing treatment. 27% of patients had a physical examination, including vitals, before starting treatment, a further 37% had just their vitals taken within 24 hours of admission and 20% refused any form of physical examination during their inpatient admission. 7% of cases had complications due to a lack of investigation.

**Conclusion:**

Although there are standard guidelines for the PICU setting, it has been noted that these guidelines aren't always implemented. Multiple factors have a role to play such as: non-consenting patients, inaccessibility of previous records, initial assessment forms being incomplete including assessment of mental capacity and lack of follow-up with physical investigations by both primary care and secondary mental health services. As per findings, a few recommendations were proposed to meet the standards.

